# Deep phenotyping to understand hearing and hearing disorders: Protocol for a feasibility study

**DOI:** 10.1371/journal.pone.0320418

**Published:** 2025-03-26

**Authors:** Ruth V. Spriggs, Paul Bateman, Raul Sanchez-Lopez, Sally K. Thornton, Olivia R. Phillips, Derek J. Hoare, Ian M. Wiggins

**Affiliations:** 1 Hearing Sciences, Mental Health and Clinical Neurosciences, School of Medicine, University of Nottingham, Nottingham, United Kingdom; 2 National Institute for Health and Care Research Nottingham Biomedical Research Centre, Nottingham, United Kingdom; LSU Health Shreveport, United States of America

## Abstract

Globally, hearing loss affects around 1.5 billion people, while tinnitus is estimated to impact around 740 million. More research is urgently needed to address the challenges presented by hearing loss, tinnitus, and other hearing-related conditions. Our plans for a Nottingham Hearing BioResource, providing research-willing volunteers and comprehensive tests of hearing and ear health repeated over time, has the potential to accelerate the field. The protocol described here is a feasibility study for this BioResource, specifically addressing questions of recruitment from the general population (i.e., outside of clinical audiology services or pathways). Participants with or without known hearing problems will be recruited for data collection. This study will quantify how feasible it will be to recruit and retain a large sample of the general population, and will suggest the demographic, and hearing condition status, distributions we could achieve for the BioResource. Data collection will involve a health and lifestyle questionnaire; cognitive assessment; five questionnaires about hearing loss, tinnitus, and hyperacusis; an estimation of lifetime noise exposure; a suite of in-depth audiological tests; and taking a hair sample. The same measurements will be taken on two separate occasions in person, and a third set of overlapping measurements will be taken remotely. Repeating the data collection will allow us to evaluate participant retention rates and establish the reliability of the measures. The findings from this feasibility study will allow us to assess which channels work well to recruit a diverse pool of participants, which, when used in conjunction with recruitment from clinic, will provide the basis for a recruitment strategy for our BioResource. In addition, we will gain useful insight into whether specific tests or questionnaires used in the feasibility study are suitable for inclusion in a deep phenotyping protocol.

## Introduction

Hearing loss is a common condition estimated to affect around 1.5 billion people globally [1]. A majority of those affected are over 50 years old, but hearing loss and related conditions, such as tinnitus, can affect people of all ages. Hearing loss is known to reduce a person’s ability to communicate effectively, worsen quality of life, and increase social isolation [1]. Tinnitus, the conscious awareness of sound without an external stimulus [2], is estimated to affect around 14% of the global population [3] and similarly can cause distress and impair quality of life [4]. Currently, there are no curative treatments available for sensorineural hearing loss [5] or subjective tinnitus [6]. Hyperacusis (reduced sound tolerance) is also common and occurs independently of hearing loss [7]. More research is urgently needed to address the challenges presented by hearing loss, tinnitus, and other hearing-related conditions, and to develop new treatments and interventions.

The Hearing theme of the National Institute of Health and Care Research (NIHR) Nottingham Biomedical Research Centre (BRC) is establishing the Nottingham Hearing BioResource. This large-scale bioresource will help accelerate research by providing a database of research-willing participants and the results of comprehensive tests of hearing and ear health repeated over time. The contents (cohorts and data) of the BioResource will be made available to other researchers, in hearing and related fields, to carry out their own research. Metadata and summary data will be available to search in a web-based tool; access to individual-level data and/or cohorts will require an application to be made. Research proposals will be assessed, using the Five Safes framework [8] as a guide, by a data access panel comprising experts, patients, and representatives from the general public.

Large-scale longitudinal health datasets, or bioresources, such as the UK Biobank [9], the NIHR BioResource [10], and the Our Future Health project [11] are powerful tools for facilitating research into the mechanisms of human health and disease [12, 13]. A limitation of existing resources is that hearing health phenotypes are captured at a rudimentary level, or not at all. For example, in the UK Biobank there are self-report questions about hearing difficulties and hearing-aid use, but objective data are limited to an automated adaptive Digit Triplet Test (DTT [14]) of speech in noise, and these data are affected by, for example, differences in ambient noise at each UK Biobank test centre [15].

The Nottingham Hearing BioResource will be unique in combining deep auditory phenotyping (comprehensive information describing individual components of the auditory system [16]) with data on noise-exposure history and genetics (through collaboration with the national NIHR BioResource [10]). Lifetime noise exposure is known to be a risk factor for hearing loss and tinnitus [17]. One key role the Nottingham Hearing BioResource could play in the future is in helping to understand how gene – environment interactions determine hearing health.

The global burden of hearing loss is increasing dramatically, and it has become the third main contributor to years lived with disability worldwide [1]. Although prevention of, and access to treatment for, hearing problems is now an urgent matter [18], the efficacy of hearing treatments and service provision requires the correct and detailed diagnosis of different profiles of hearing loss and the discovery of distinct auditory phenotypes. Hearing loss is complex and there are a range of mechanisms of impairment that can lead to similar audiometry test results [19]. Identifying a specific cause for hearing loss, or a phenotypic subtype, is becoming more important with the advent of emergent auditory therapeutics [20, 21] that will target specific components of the auditory system, and therefore benefit subsets of patients.

Participants in the Nottingham Hearing BioResource, with and without hearing or ear problems, will undergo a series of in-depth tests to measure different aspects of hearing and ear health. Participants will also complete questionnaires to provide information about their hearing and general health, and provide samples such as hair and blood for biomarker and genetic analyses. The aim of the BioResource is to follow participants as they age, by periodically repeating tests and questionnaires, and adding new tests as necessary, to allow researchers to link together different aspects of data, identify long-term patterns (e.g., whether lifestyle changes can reduce a person’s susceptibility to experiencing tinnitus), and identify distinct groups of patients with similar phenotypes. Our aim is to leverage the power of large, accessible datasets in a way that can transform how we treat and manage hearing loss and hearing-related conditions in the future; a future where the population is ageing and the need for effective treatments is set to increase [22].

The protocol described here is for a feasibility study; a sample of participants will be recruited and tested to ensure that the core processes put in place, including recruitment channels, audiological test pipelines, and follow-up scheduling, are all working effectively. The population intended to participate in this research will be adults with or without hearing loss, tinnitus or any other hearing condition. We are not targeting any particular hearing conditions in our recruitment for the feasibility study. Efforts will be made to ensure that recruitment occurs across a broad spectrum of the population and results will be analysed with respect to this.

For the feasibility study, a subset of audiological tests will be used, and only samples of hair will be taken. Hair samples are requested to gauge participant willingness to provide such samples for biomarker analysis. Participants will be tested on recruitment, re-tested after three months, and asked to repeat questionnaires and do an online listening task three months after the re-test. Repeating the audiological tests after three months allows the reliability of these tests to be established so that meaningful changes in the results, after longer time periods, can be identified. Asking participants to be involved on three separate occasions also allows us to assess participant willingness to maintain involvement with the BioResource, in-person and remotely, over time. It is envisaged that supplementary remote data collection will play an important role in the future BioResource, particularly in relation to longitudinal follow-up. Among the tests to be performed are routine clinical tests, but also novel tests including the wideband middle ear muscle reflex test [23] (MEMR), a non-invasive test that has been proposed as a marker of auditory nerve degeneration (cochlear synaptopathy). Data from this study will support or refute the future inclusion of this test in the BioResource.

The overall aim of the Nottingham Hearing BioResource we are establishing is to develop a flexible resource containing longitudinal deep phenotyping data that can be used to support research into a broad range of hearing, and hearing-related, conditions/disorders. This feasibility study will quantify how well we are able to recruit, test, and retain participants, at the scale required to create a bioresource that is genuinely useful to researchers working in hearing and related disciplines. We will measure the rates of recruitment and retention, as well as data completeness, the range of times taken to carry out baseline and second appointments, and the ratio of participants with hearing loss and/or other hearing conditions to those without. This study will also provide initial data to use for developing robust data handling and analysis pipelines, and information on the reliability of individual tests.

## Materials and methods

### Aim, design and setting

This is a feasibility study recruiting from the general population in the East Midlands region of the United Kingdom. The primary aim of this study is to evaluate; the feasibility of administering a large audiological test battery (deep phenotyping, as detailed in the Processes and measures section below) in adults with and without hearing disorders, how well we can recruit participants to take part, and whether we can retain them for repeated testing.

The primary objectives are to evaluate: the recruitment and retention rates achieved through the tested recruitment channels, the data and sample completeness achieved and the time taken for each appointment, and the distribution of hearing conditions and demographics recruited through each channel. How we will use these metrics to evaluate feasibility is described in the Statistical analyses section below. We hypothesise that the test battery and repeated assessments will be acceptable to the general population.

The secondary aim of this study is to determine the test-retest reliability and co-variability of our test battery of audiological phenotyping tests. The objectives are: to calculate test-retest metrics (as described in the Statistical analyses section below) when audiological tests and questionnaires are carried out 3 months apart, and to investigate any correlations between the results of different audiological tests and other collected measures.

### Participants

All adults will be eligible to participate (subject to the exclusion criteria below). Detailed information about each participant’s self-reported hearing loss and other hearing-related conditions, or lack thereof, will be collected and analysed, but do not form part of the inclusion criteria. Participants without known hearing-related issues will be needed in any future BioResource to form a control group.

#### Inclusion criteria.

- Able to give informed consent
- Aged 18 or over
- Able to attend 2 in-person appointments with a duration of 2 hours each

#### Exclusion criteria.

- Actively discharging ear infections
- Malformation of the ear such that headphones or ear inserts cannot be used
- Hearing intervention fitting or surgery on any part of the audio-vestibular system within the last 2 months
- Concurrent participation in hearing intervention research
- Fitted with a programmable ventriculo-peritoneal shunt

#### Sample selection.

Participants will be recruited through social media posts, advertising in private sector audiology settings, public engagement at local events, and through the NIHR Nottingham BRC Hearing Theme volunteer database of people who have expressed an interest in taking part in research*.* This will provide evidence of the potential demographic distribution of cohorts recruited in these ways and what the recruitment and retention rate could be for our BioResource.

Participants will receive an inconvenience allowance of £10, and up to £15 travel costs, for each in-person appointment.

#### Sample size.

We have set a pragmatic target of 84 participants. This is equivalent to approximately 2 months recruitment into the future BioResource based on a projection of recruiting 2500 participants over 5 years. Recruitment will continue for up to 12 months until 84 participants have been recruited. This study does not require any grouping of participants, or any randomisation or blinding.

#### Consent.

Eligible participants will be identified from volunteers who contact the study team. Participants will be given an Information Sheet detailing their rights and what will happen during the study, and will be asked to provide written informed consent. Consent will be re-confirmed at each data collection session.

### Processes and measures

All measures were selected for inclusion in the protocol based upon iterative discussions across a panel of hearing scientists at the University of Nottingham. The length of involvement and the contents of the feasibility study were also considered by a panel of three Patient and Public Involvement (PPI) representatives.

**Fig 1:**
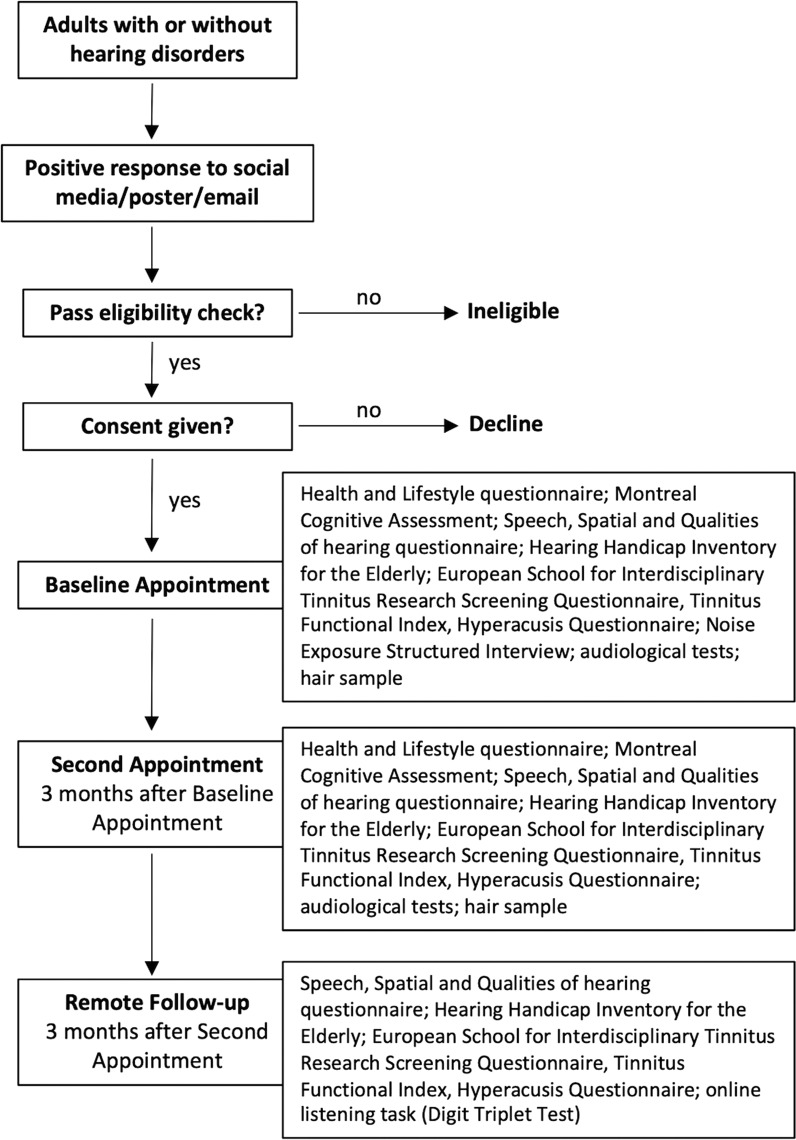
Participant Journey. Flowchart showing participant’s journey in the feasibility study, from volunteering through to completing the study.

During the feasibility study, the same measurements will be taken from all participants on two separate occasions (in person) and a separate overlapping set of measurements will be taken from all participants on one occasion (remotely) (Fig 1). No interventions are made; this is a study concentrating on technical and operational feasibility.

#### In-person appointments.

At the in-person appointments, participants will first complete a health and lifestyle questionnaire, and provide a self-report of any hearing-related issues, tinnitus, or treatment for hearing loss. The Montreal Cognitive Assessment (MoCA) [24] will then be performed (using the hearing impaired version if necessary (MoCA-H [25]). This test produces a score to indicate potential cognitive impairment. This will be followed by the completion of 5 Patient Reported Outcome Measures (PROMs):

- Speech, Spatial and Qualities of hearing questionnaire (short version; SSQ12) [26]
- Hearing Handicap Inventory for the Elderly (HHIE) [27]
- European School for Interdisciplinary Tinnitus Research Screening Questionnaire (ESIT-SQ) [28]
- Tinnitus Functional Index questionnaire (TFI) [29]
- Hyperacusis Questionnaire (HQ) [30]

The Noise Exposure Structured Interview (NESI) [31] will be administered by the researcher to assess the extent of the participant’s noise exposure during their lifetime. The NESI will only be carried out at the first appointment.

Video otoscopy will be performed to ensure it is safe to proceed with audiological testing. Participants will complete the following audiological tests, in each ear, in a soundproofed booth:

- Pure tone audiometry (250, 500, 1000, 2000, 3000, 4000, 6000 & 8000 Hz) [32]; masking will be used if necessary
- Bone conduction audiometry (500, 1000, 2000 & 4000 Hz) [32]
- Extended high-frequency audiometry (9000, 10000, 11200, 12500, 14000 & 16000 Hz) [33]
- Quick Speech In Noise test (QuickSIN) [34]
- Wideband Tympanometry (WBT) [35]
- Wideband Middle Ear Muscle Reflex (MEMR) test [23]
- Otoacoustic Emissions (OAE) [36]; Transient Evoked (TE) and Distortion Product (DP)
- Threshold Equalising Noise (TEN) test [37]

A small sample of hair will be taken (where possible) from the posterior vertex of the head as close to the scalp as possible (see demonstration at https://youtu.be/jWt_XWlcl5Y) and the samples processed (using a 3 cm segment from the scalp-near end) using commercial assays and standard protocols to measure cortisol levels over the previous 3 months [38, 39]. Cortisol levels are an indicator of stress and have been suggested as a useful biomarker for use in hearing research, for example, to evaluate the efficacy of an intervention [40, 41].

The complete test battery (except NESI) will be repeated at a second appointment 3 months after the first appointment. More details concerning the 5 PROMs and the battery of audiological tests listed above can be found in Table 1.

**Table 1:** Brief descriptions and test-retest information for phenotyping measures.

Name of measure	Brief description	Example test-retest results
Speech, Spatial and Qualities of hearing questionnaire (short version; SSQ12)	12-item version of the complete 49-item SSQ measuring self-reported disabling effects and impacts of a hearing deﬁcit.	No test-retest results published.
Hearing Handicap Inventory for the Elderly (HHIE)	Self-assessment tool to assess the emotional and social effects of hearing impairment.	Test-retest reliability; 95% confidence interval = 36.0% using paper-and-pencil format independent of clinician [42]. Scores highly correlated (r = 0.84 [95% CI, 0.70-0.93]) [43].
European School for Interdisciplinary Tinnitus Research Screening Questionnaire (ESIT-SQ)	Self report instrument for potential risk factors for tinnitus and tinnitus characteristics.	No test-retest results published.
Tinnitus Functional Index questionnaire (TFI)	Self-report questionnaire for scaling the severity and negative impact of tinnitus.	Test–retest reliability 0.78 (r) [29].
Hyperacusis Questionnaire (HQ)	Self-report questionnaire to quantify and evaluate hyperacusis symptoms.	No test-retest results published.
Pure tone audiometry (air conduction PTA)	Standard procedure used in audiometry clinics to assess hearing thresholds at varying frequencies.	Good test-retest reproducibility. E.g., ≤ 10 dB variability in 100% of participants at 1, 3 and 4 kHz and in 97% of participants at 6 kHz [44], test-retest repeatability within 10 dB for > 99% of measurements from 0.5 to 6 kHz [45], and mean dB HL difference under 5 dB at all frequencies [43].
Bone conduction audiometry	Standard procedure used in audiometry clinics to assess hearing thresholds at varying frequencies using bone conduction to bypass the outer/middle ear.	Test-retest threshold difference of 5.1 dB (5.3 SD) [46]. Good test-retest reliability; mean dB HL difference under 5 dB at all frequencies [43].
Extended high-frequency audiometry	Pure tone audiometry at frequencies higher than 8 kHz.	Good test-retest reproducibility. E.g., within 2 dB at all frequencies [47], and test-retest repeatability within 10 dB for 94% of measurements at 8 to 16 kHz [45].
Quick Speech In Noise test (QuickSIN)	Measures the SNR (signal-to-noise ratio) a listener requires to understand 50% of key words in sentences in a background of babble.	Estimated accurate to +/-2.7 dB at the 95% confidence level. Test-retest standard deviation of 1.4 dB SNR for hearing-impaired [34]. Correlation between scores not significant (r = 0.34 [95% CI, − 0.08 to 0.68]) [43].
Wideband Tympanometry (WBT)	Measures middle ear function using wideband acoustic immittance under varying ear-canal pressures.	Test-retest reliability excellent (immediate test–retest without reinsertion of the probe), for comparison statistics see papers [48, 49].
Wideband Middle Ear Muscle Reflex (MEMR) test	Changes in MEMR amplitude (bilateral contraction of the middle ear stapedial muscle in response to moderate-to-high intensity acoustic stimuli) have been proposed as an assessment for noise-induced synaptopathy.	High test-retest reliability when elicitor stimuli presentation levels are between 70 and 90 dB SPL (moderate reliability at 60–65 dB SPL, low at 45–50 dB SPL). For details, see [50].
Otoacoustic Emissions (OAE); Transient Evoked (TE) and Distortion Product (DP)	OAEs are sounds of cochlear origin produced by sensory outer hair cells in response to auditory stimulation, and are used to test the function of the middle and inner ear. TEOAEs are evoked by a broadband click. DPOAEs are evoked by two simultaneously presented pure-tones.	Overall, test-retest reliability of TEOAEs and DPOAEs is high, but reliability decreases after probe-refitting and with longer time intervals (figures in [51]). Test–retest reliability of DPOAE is good at 1.5 kHz (ICC = 0.78, 95% CI 0.48 to 0.91) and excellent at 4 kHz (ICC = 0.91, 95% CI 0.76 to 0.97) [50]. DPOAE: percentage agreement high, ranging from 78% to 100% [43].
Threshold Equalising Noise (TEN) test	The TEN test is used to find cochlear dead regions by measuring pure tone thresholds with a specific (TEN) masking noise.	High test-retest repeatability (meeting criteria for DR 97%, identifying same edge frequency 87%) [52].

#### Remote follow-up.

This session will take place 3 months after the second appointment. Participants will complete an online form containing the 5 PROMs (SSQ12, HHIE, ESIT-SQ, TFI, HQ) and an online version of the DTT [14]. Participants unable to access online tools will be offered a telephone call or postal versions of the questionnaires and will omit the DTT.

#### Participant viewpoint.

At the in-person appointments, participants will additionally be asked hypothetical questions as to whether they would be willing to join the Nottingham Hearing BioResource, whether they would be willing to give a blood sample to take part in that BioResource, and whether they would be happy for that blood sample to also contribute to the national NIHR BioResource [10].

After each stage of the study, participants will be asked about their satisfaction with the process, whether they have any suggestions for improving the participant experience, and whether they have any reservations about continuing in the study. Participants that fail to attend their second appointment or to complete the remote online session will be given the opportunity to provide their reasons why and these will be documented.

### Data management

Data collection throughout the study will be continually monitored and analysed to ensure quality and to monitor the efficiency of different recruitment approaches. Reasons for missing data will be logged (e.g., a test may be abandoned because the participant is uncomfortable) and the reasons will be analysed to determine improvements to the data collection process for the BioResource.

A data management plan is in place. Data will be securely collected and managed using the REDCap [53] electronic data capture tool hosted at the University of Nottingham, and stored in a MySQL database [54]. Personal information will only be accessible to researchers administering the study. Data exported for analysis by the research team will be de-identified.

Data from the feasibility study will be made available to external researchers on request. The data will be findable through a Digital Object Identifier (DOI) in the Nottingham Research Data Management Repository [55], providing metadata and instructions on how to request a dataset. Data shared with external researchers will be pseudonymised (with keys unique to each extraction) and de-identified before sharing.

### Safety considerations

There are minimal risks to the participants taking part in this study; all tests are minimally invasive and have no known side effects. There is a chance of incidental findings, such as undiagnosed hearing loss, and affected participants will be advised to contact their General Practitioner.

### Statistical analyses

#### Primary aim.

Descriptive and simple comparative statistics (such as t-tests/ANOVA or χ^2^, as appropriate) will be used to: compare recruitment rates between channels; evaluate retention rates to second appointment and remote session, data and sample completeness, and time taken for each appointment; and assess the distribution of hearing conditions and demographic characteristics recruited through each channel.

The feasibility of recruiting into the Nottingham Hearing BioResource through the tested channels will be assessed based on their relative recruitment rates (mean number of participants per month), the resulting retention rates, and the diversity of the samples recruited. We are aiming for 80% retention to the end of the study; a figure lower than 50% will decrease our ability to maintain a usefully longitudinal BioResource indicating that we may need to put in place extra measures to increase participants’ long-term interest. The distribution of participants in terms of hearing health and other demographic features will give an indication of how broad and balanced a dataset can be achieved with our current recruitment methods and allows us to plan improvements where necessary. The BioResource needs to recruit participants that reflect the wider population they are recruited from in order to benefit everyone in that population. We also need to capture a wide range of people with different hearing disorders to enable stratification of those hearing disorders based on phenotypic descriptors.

We will also look at the success of the testing process; if a section of the protocol has a high rate (>50%) of incomplete data, its future inclusion in the BioResource may be revised or reconsidered. With respect to the time taken to complete the in-person data collection, we are aiming for 1.5 hours (to allow the overall appointment to take no longer than 2 hours); if the distribution of times is skewed towards > 1.5 hours, action may need to be taken to reduce this (taking into account participants’ satisfaction with the process).

#### Secondary aim.

Intraclass correlation coefficients with 95% confidence intervals and Bland–Altman plots will be used to assess the test-retest reliability of the audiological phenotyping tests and PROMs performed 3 months apart. These test-retest metrics will be compared to previously published information about the reliability of these tests (see Table 1) to assess the accuracy and reliability of these tests in our hands. Where no test-retest results have been previously published, for example, for SSQ12, our results will add to the field. Establishing the possible magnitude of the difference in results when tests are repeated soon after each other allows meaningful changes in results, when tests are repeated over longer time periods, to be identified.

Spearman’s rank or Pearson’s correlation coefficients and regression analysis, as appropriate, will be used to investigate relationships between different audiological tests, between audiological tests and PROMs, and between tests/PROMs and hair cortisol levels. To perform these analyses, audiological tests and PROM questionnaires will be converted into final scores as appropriate, for example, pure tone audiometry results will be converted into better/worse-ear across-frequency averages, and the SSQ12 produces an overall score out of 10. Consistent correlations between measures may inform the inclusion/exclusion of redundant measures in the deep phenotyping protocol used in the Nottingham Hearing BioResource. No analyses will be performed for the feasibility study to explicitly compare measures between participants grouped by hearing loss/hearing condition status, but this will be considered as a potentially confounding factor when looking at correlations between measures.

### Status and timeline

Recruitment and enrolment began on 7^th^ October 2024 and will cease after 12 months (6^th^ October 2025) or when 84 participants have been recruited. Participants will be involved in the study for approximately 5 hours over a maximum of 7 months (the second appointment is within 3 months of the start and the remote follow-up is within 3 months of the second appointment, both within a 2-week window).

### Ethics

This study has been given a favourable opinion (FMHS 216-0624) by the University of Nottingham’s Faculty of Medicine & Health Sciences Research Ethics Committee. Written informed consent will be sought from each participant before they enter the study.

Ongoing review of the protocol and its implementation during the feasibility study will be crucial to promptly address any challenges and identify where changes to the protocol may be needed. Any protocol changes will be made with the involvement of the PPI representatives and in accordance with research ethics procedures.

## Discussion

We describe a protocol for a study to evaluate the feasibility of a Nottingham Hearing BioResource to deeply phenotype hearing and hearing disorders. Feasibility will be determined according to recruitment and retention rates through different channels targeting the general population, data completeness, and the diversity of participants in terms of demographics and hearing health. This study will also provide initial data to use for developing robust data handling and analysis pipelines, and to evaluate test-retest reliability and correlations between phenotyping measures.

In the longer term, we will also be recruiting through clinical pathways into the BioResource, but we will need to recruit from the general population to ensure we get a diverse cohort with and without hearing disorders. Therefore, this study tests general population recruitment channels only. Participants in the feasibility study will be given the opportunity to join the BioResource and future studies/data collection.

More research is urgently needed to address the challenges presented by hearing loss, tinnitus, and other hearing-related conditions, and a Nottingham Hearing BioResource, providing research-willing volunteers and comprehensive tests of hearing and ear health repeated over time, has the potential to greatly accelerate this.
